# University Students' Knowledge and Perceptions About Concepts, Recommendations, and Health Effects of Added Sugars

**DOI:** 10.3389/fnut.2022.896895

**Published:** 2022-06-09

**Authors:** Isabela Paz Santana, Tailane Scapin, Vanessa Mello Rodrigues, Greyce Luci Bernardo, Paula Lazzarin Uggioni, Ana Carolina Fernandes, Rossana Pacheco da Costa Proença

**Affiliations:** Nutrition in Foodservice Research Center (NUPPRE), Nutrition Postgraduate Program (PPGN), Federal University of Santa Catarina (UFSC), Florianópolis, Brazil

**Keywords:** sugary drinks, focus group, sugary foods, food labeling, healthy eating

## Abstract

It is recommended to limit added sugars to below 10% of the daily energy intake, as excessive consumption has been associated with several chronic non-communicable diseases. This exploratory qualitative study used focus groups to investigate the knowledge and perception of Brazilian university students about added sugars concepts, consumption recommendations, and health effects. Focus groups were led by a moderator using a semi-structured discussion guide. The focus groups were recorded, transcribed verbatim, and subjected to thematic analysis. Five focus groups were conducted with a total of 32 participants (50% women, mean age 23 years). Participants could not distinguish added sugars from sugars naturally present in foods and were unaware of the health impacts associated with excessive added sugar consumption, except for the risk of diabetes. Although most participants reported limiting sugar consumption, they had no knowledge of official consumption recommendations. Given that current public policy agendas aim to reduce added sugar intake, there is a need to strengthen strategies for disseminating information on added sugar concepts, recommendations, health effects and how to identify them in the foods products.

## Introduction

Sugars, chemically defined as monosaccharides and disaccharides, are included in the group of carbohydrates, together with starch and dietary fibers (1). Sugars can be classified as intrinsic sugars, which are naturally present in fruits, vegetables, and milk, and as added sugars, which include all types of sugars added to foods and beverages during processing, meal preparation, or at the time of consumption (2, 3). The term “free sugars” is often used to refer collectively to added sugars and sugars naturally present in honey, syrups, and fruit juices (4). The term “total sugars” refers to all types of sugars.

There is no evidence indicating harmful health effects associated with the consumption of intrinsic sugars. In contrast, several studies have been demonstrating association of excessive added sugar consumption and development or worsening of non-communicable diseases, such as hypertension, cardiovascular diseases, and dental caries (5–10). In 2015, the World Health Organization (WHO) published dietary guidelines recommending limited intake of free sugars to not exceed 5–10% of the total daily energy intake (4). Similarly, national dietary guidelines, such as the Dietary Guidelines for the Brazilian Population (11) and the US Dietary Guidelines (12), recommend limiting the consumption of foods and beverages containing high amounts of added sugars.

Despite such recommendations, global food supply data from 2000, 2006, and 2013 demonstrated that there has been an increase in sugar availability, mainly through sugary drinks. In addition, the Latin America region has the second highest consumption of sugary drinks per capita, with Brazil ranking among the top countries in sugary drink sales (13). Data from a budget survey conducted in Brazil between 2017 and 2018 with a nationally representative sample showed an elevated consumption of foods and beverages high in added sugars, particularly of ultra-processed foods (14). In addition, a cross-sectional study based on data from the Brazilian National Health Survey (NHS), conducted in 2013/2014 has demonstrated that young adults are the main consumers of sweet foods and soft drinks (15).

Although it is known that high consumption of added sugars is a worldwide problem and global recommendations for limiting added sugars have been in place since 2015, there is scarce scientific discussion on the topic. The few studies that have examined consumers' understanding and perceptions of added sugars demonstrated lack of understanding about sugar concepts, consumption, and recommendations (16–22). No studies conducted with Brazilian consumers were found. The investigation of perceptions regarding added sugar is particularly important among university students, since they are a subgroup of young adults with particular characteristics. They are usually in a phase of transition characterized by the development and consolidation of new habits and behaviors, including those related to food consumption (23). A review study showed that most university students have unhealthy eating behaviors, including high intake of fast foods, snacks, sweets, soft drinks, and alcoholic beverages and low intake of fruits and vegetables (24, 25).

Exploratory qualitative research can help to understand consumers' perceptions of issues related to sugar intake and thus guide the development of targeted strategies to limit added sugar consumption by specific populations. Considering the excessive intake of added sugars by the Brazilian population, particularly by young adults, and the lack of studies on the topic, this study aimed to investigate the knowledge and perceptions of university students about the concepts, consumption recommendations, and health effects of added sugars.

## Methods

### Study Design and Participants

This qualitative investigation used the focus group method to examine the perception of university students about added sugars attitudes. The study was conducted with a convenient sample of university students from a major university located in a South capital city in Brazil. The targeted university has more than 30,000 undergraduate and postgraduate students enrolled in more than 100 on-campus courses. Students are from all parts of Brazil with different socioeconomical status.

Participants were recruited through printed and online posters containing information about the survey, an electronic address, and a QR code linked to an online questionnaire. The questionnaire was used to collect sociodemographic, anthropometric, and health data as well as information on time availability for participation in the study. A screening question about the use of food label was included. Only university students who reported paying attention to food labels were invited to participate. The printed posters were spread through the university campus close to areas with a high flow of students such as the library, the convention center, and the cafeterias. The online posters were published on the social medias of the research group. All students had equal access to the advertising materials, although the online posters were more likely to be viewed by health students than students from other schools.

The following eligibility criteria were used: (i) undergraduate and/or graduate university student aged 18 years and older, (ii) not enrolled in nutrition courses or holding a nutrition degree, (iii) not being part of the research study group, (iv) willingness to participate in the study and signing an informed consent form, and (v) completing the online recruitment form and appearing in person on the day of the focus group. Participants were contacted *via* e-mail and telephone to confirm their availability and schedule the face-to-face meeting. During recruitment, participants were asked to share recruitment information with friends and colleagues who met eligibility criteria without mentioning the content of the discussion, as a form of snowball sampling.

### Focus Groups

Focus groups were conducted in Portuguese between May and June 2019. Each focus group had a minimum of 4 and a maximum of 10 participants, and sessions lasted up to 75 min. The endpoint of data collection was defined as the point of idea saturation, when new thoughts were not emerged by the participants. Focus groups were facilitated by one investigator (TS) supported by a research assistant (IPS). The investigator explained the importance of all participants contributing to the discussion and emphasized that there were no correct answers. All discussions were audio-recorded.

Focus groups were conducted using a semi-structured guide containing short open questions in an easy-to-understand language. Questions were designed to examine students' understanding of added sugar concepts, consumption recommendations, and health impacts. Some encouraging sentences (probes) were included between questions to stimulate discussion (e.g., “why?” “how?” “tell us more about it”). Prior to focus groups, the discussion guide was tested for clarity of wording and meaning with experts and members of our research group.

### Data Processing and Analysis

Recorded audios were transcribed verbatim using *Speechnotes* and imported into MAXQDA® for analysis. Thematic analysis was used for extraction of codes, categories, and central themes (26). Three triangulation procedures were used to ensure data reliability. In the first triangulation, a second researcher independently coded 10% of the data, and the agreement between codes and themes was assessed. In the second triangulation, codes and categories were discussed with two researchers experienced in qualitative research and who have participated in the design of the survey. Finally, as a strategy to increase reliability, we used direct quotations from participants to illustrate the identified themes and conclusions.

### Ethical Aspects

We obtained ethical approval from the Human Research Ethics Committee of the Federal University of Santa Catarina, Brazil (Protocol No. 3.063.750). All participants involved in this study gave written informed consent before participating. Consent was confirmed verbally before recording devices were turned on during focus groups.

## Results

Eighty university students filled in the recruitment questionnaire. All of them were contacted at least twice by the researchers to schedule a suitable time for participating in the focus groups. From the 80 respondents contacted, 32 (40%) were available to attend an in-person meeting. The 32 students were spread into five focus groups according to their availability. Overall participants' mean age was 23 ± 4.1 years, half of them were female and 75% were undergraduate students from different courses, such as health sciences, economics, engineering, and biology. Most participants (94%) did not report a diagnosis of chronic non-communicable diseases, and 22% reported dietary restrictions (mostly lactose intolerance). Table 1 shows the participants' characteristics according to each focus group. No significant differences in participants' mean age and BMI between focus groups were found, demonstrating homogeneity across the groups.

**Table 1:** Focus group participants' characteristics.

	**Focus group 1**	**Focus group 2**	**Focus group 3**	**Focus group 4**	**Focus group 5**
Number of participants	8	6	4	5	9
Age mean years (SD)	24 (4.6)	25 (4.0)	22 (7.2)	22 (2.1)	21 (2.3)
% of female	75%	50%	50%	60%	33%
% of undergraduate students	75%	33%	75%	80%	100%
BMI mean kg/m^2^ (SD)	23.4 (2.3)	24.3 (4.0)	21.5 (2.2)	25.3 (4.5)	24.5 (2.9)
% of people with dietary restrictions	22%	50%	25%	60%	11%

Thematic analysis of focus group transcriptions revealed four major themes: (i) characterization and differentiation of types and sources of sugars, (ii) confusion about the concept and metabolism of sugars and carbohydrates, (iii) unawareness of recommendations for sugar consumption, and (iv) negative health impacts of sugar consumption.

### Characterization and Differentiation of Types and Sources of Sugars

The participants associated sugars mainly with sensory characteristics, such as sweetness and tastiness, and their role as energy sources. Many students reported not knowing how to distinguish “natural” sugars from those added to foods and they also demonstrated difficulty in conceptualizing the different types of sugars. Students had different perceptions of sugar types and consumption sources. Packaged foods, particularly soft drinks, were cited as sources of sugars. Whereas some participants identified differences between sugars from a chemical point of view, others mentioned that there are differences between natural sugars and those present in packaged foods. Some participants had low or no knowledge about the differences between added and naturally occurring sugars.

“*I know that some sugars have different names, so we often can't identify them. This kind of deceives the consumer, using large chemical names so we won't know what sugar is. I know these little tricks. (Focus group 4, female)”*

All focus groups discussed the different types of sugars found in foods. Some participants considered that fruit sugar is the best type of sugar. Honey was deemed to be as natural as fruit sugar. According to some participants, sugars differ in nutritional quality. Brown and organic sugar were reported as being nutritionally better than refined sugar, although price was cited as a major determinant of purchase intention. As the discussions progressed, students showed interest in reducing sugar consumption, particularly that of sugars added to homemade foods.

“*I've tried several types of sugars to see what they're like. Price is also important; organic and brown sugar are much more expensive. I prefer brown sugar for its color. I read that the darker, the healthier. (Focus group 2, male)”*

### Confusion About the Concept and Metabolism of Sugars and Carbohydrates

In some focus groups, participants associated the concept of sugars with that of carbohydrates. Some seemed to be confused about this point, assuming that all carbohydrates are sugars. Others believed that sugars were correlated with carbohydrates but could not clarify the relationship between the two components.

“*That's a doubt I have. Sometimes it's written [on food labels] sugars and carbohydrates. Yeah, but aren't carbohydrates sugars?… For me, carbohydrates are sugars. (Focus group 5, male)”*

Some participants have also stated that sugars are metabolized differently in each organism, which is why they consider it difficult for some people to understand how much sugar they need to eat. Students believed that many people do not understand the relationship between consuming foods that contain carbohydrates and the provision of sugars to the body for energy supply.

“*About this issue with carbohydrates… because people eat carbohydrates. For example, bread. People eat bread but they don't know that they're eating sugar. People eat pasta and they don't know that they're consuming sugar. So how many diabetics eat these foods unaware? (Focus group 5, female)”*

### Unawareness of Recommendations for Sugar Consumption

Most of the participants reported that consuming less sugar is better for health. Some mentioned that they have been trying to reduce daily sugar consumption, especially from the addition of sugars in homemade foods. Despite this concern, participants could not specify the amount of sugars they considered suitable for consumption. When participants were asked about how much sugar can be consumed as part of a healthy diet, most of them remained silent or generally answered “I do not know.”

“*I imagine that there is some recommendation for consumption [of sugars] but I have no idea how much it is (Focus group 3, female).”*

In some focus groups, however, students cited values that were close to WHO thresholds. Some considered that sugar limits depend on the level of physical activity and/or individual energy expenditure. Only one student reported the WHO daily recommendation for sugar intake.

“*I think it depends on a person's energy expenditure. If the person performs physical activity, they will need more carbohydrates, right? (Focus group 4, male)”*

“*I have a mobile app that tells me my ideal sugar intake. It says 47 g, almost 50 g. But it's just an app; it's not a reliable source, right [laughs]? (Focus group 5, male)”*

### Negative Health Impacts of Sugar Consumption

Participants associated excessive sugar consumption with negative health effects, particularly the development of diabetes. Other chronic diseases, such as obesity, cancer, and atherosclerosis, were also mentioned, as well as dental and thyroid problems. In addition, the relationship between sugar consumption and anxiety, stress, and depression was something that has emerged in all focus groups. According to some participants, consumption of high-sugar foods is common under these situations and may momentarily relieve tension.

“*I rarely eat refined sugar. But at the end of the semester, I usually have a sweet tooth… I feel more like eating sweets when I'm stressed. (Focus group 1, male)”*

Finally, few participants reported their perception that sugar consumption may lead to addictive behaviors and because of that is a nutrient that should be eaten with attention.

“*Especially in periods of high demand, I like to eat a lot of sugar; if not, it feels like my body can't relax. So, I think it's an addictive thing and it's hard to stop. (Focus group 2, male)”*

## Discussion

The results of the present study indicate that most participants could not distinguish intrinsic sugars from added sugars and deemed that the type of sugar (brown vs. refined) was more important than the fact of being added or intrinsic. Participants considered that sugar consumption can cause dependence and that excessive consumption is associated mainly with diabetes development and, less frequently, with other health problems such as obesity and tooth decay. In general, participants were unaware of sugar intake recommendations.

In the present study, participants identified sugars as energy sources. Similar results were observed in a qualitative study conducted in Australia, in which participants reported that high consumption of sugary drinks was associated with trying to prevent an energy deficit (17). The Australian study also showed that participants reported knowing that sugary drinks were not healthy but, nevertheless, found that daily consumption of these beverages was frequent and normal. A similar perception was expressed by the Brazilian university students who have participated in the present study; they mentioned consuming sugars regularly despite knowing the harmful effects of excessive sugar intake.

Participants reported that packaged foods, particularly soft drinks, contain high amounts of sugars. In a study in South Africa, consumers considered fruit-based soft drinks to be high in sugar but frequently consumed these products, mainly because of low prices, marketing strategies, and personal preferences (27). Participants also mentioned interest in reducing sugar intake, particularly from homemade foods. According to the 2017–2018 Brazilian Consumer Expenditure Survey, the Brazilian population has reduced sugar consumption: the purchase of crystal and refined sugars decreased by 50 and 40%, respectively, from 2002 to 2018 (14). These data suggest a trend in the reduction of sugar consumption at home, both at the table (added to juice, tea, and coffee) and in cooking (as an ingredient of cakes and desserts). However, packaged food consumption has increased, and these foods are high in added sugars (28, 29).

Study participants cared about the type of sugars, as they valued brown, organic, and fruit sugars instead of refined sugar. This perception is consistent with the results of a study conducted with adult consumers in Switzerland, which showed that using the label “fruit sugar” instead of “sugar” in packaged foods increased participants' perceived healthiness of the foods (30). Regarding the production type, a study analyzing adult consumers' perception of organic foods in Brazil showed that these products are perceived as healthier than the conventional and as they can improve the quality of life of the consumers (31). Other study conducted with Brazilian university students showed that food healthiness was associated with being natural and containing low amounts of additives (32), which can explain why the participants in our study valued organic sugar.

In the current study, participants demonstrated confusion or unawareness of the concepts of sugars, not being conscious about the types of sugars. This lack of information was also observed in a qualitative focus group study on the understanding of US consumers about sugar labeling. The US participants reported that the presence of added sugar labels indicated that food contained more sugar than usual, added by the manufacturer, making the product less desirable (33). A lack of understanding of these concepts is not restricted to consumers; it can also be observed in the scientific literature. An analysis of studies discussing the topic revealed a diversity of terms used to refer to sugars (including free sugars, added sugars, extrinsic sugars, and total sugars) and difficulty in reaching a consensus on definitions (3, 34). Many participants mistakenly related carbohydrate as a synonym for sugar. This finding agrees with those of a study conducted with 940 mothers in Taiwan: half of the subjects could not determine the sugar content of a food product because they did not know the difference between sugars and carbohydrates (35).

Many participants in the present study associated sugar consumption with pleasure or reward, often as compensation for hard work or physical exercise. Similarly, university students from the Emirates related added sugar consumption to emotional factors, stating that they consume more added sugars when feeling stressed (36). The perception that sugar can cause addiction was also emerged by some participants in our study. These results are similar to those found in a qualitative study with university students from Portugal, where sugar was simultaneously perceived as pleasurable and needed, but also as addictive and harmful (18). Some evidence has shown that adults present withdrawal symptoms after cessation of prolonged sucrose consumption, with behaviors similar to those of depression and anxiety (37–39).

Although WHO recommendations for free sugar consumption were published in 2015, 4 years before our data collection, only one student was able to accurately report the official values, demonstrating that almost all participants had no knowledge of the topic. Similarly, in a study conducted in Taiwan with 122 mothers, 40% of participants with high education could not inform WHO sugar recommendations, even after receiving face-to-face and online training on the topic and the proportion reached 80% when considering those who received online training only (40). These findings indicated that official consumption guidelines have a limited reach and are difficult to understand, which may lead to excessive or poorly informed sugar consumption. Although participants in our study were mostly unaware about the recommendations for sugar intake, they have mentioned some harmful effects of excessive intake of sugars. The development of diabetes was the most cited example of harmful effects associated with high sugar consumption, followed by obesity and tooth decay. In contrast, university students from Portugal have demonstrated few concerns about harmful effects of excessive consumption of added sugars by young adults and were mostly concerned about the negative impact of high sugar intake on body image (18).

Some limitations of this study need to be considered. We conducted a voluntary survey and because of the topic of the survey it is possible that individuals with more health concerns have been volunteered. Nevertheless, during focus groups, participants seemed to be unaware or confused about sugar consumption recommendations and they have demonstrated mixed feelings about health concerns. Another limitation is that the results of this study are not representative of individuals in other contexts, with different socioeconomic status, or from other parts of the country. However, care was taken in ensuring heterogeneity among participants regarding sex, field of study, and age, excluding students from nutrition courses. Nevertheless, future studies with students from other universities or with young adults out of the university environment are needed to produce a broader perspective and representativeness of the findings to the whole age group. In addition, we recommend that future studies investigate the source of nutritional information that students use to make their foods choices and what are their perceptions about artificial sweeteners, the main sugar alternative. Finally, the findings of this study can be used for planning large scale surveys or interventions investigating the topic in a bigger sample, as well as to subsidize discussions around interventions aiming to lower the sugar intake in the Brazilian population.

## Conclusion

The current results demonstrate unawareness about the types of sugars and lack of knowledge about the recommendations for added sugar consumption among a sample of Brazilian university students. Some students considered carbohydrates to be synonyms for sugars or to have equal metabolic effects. Sugars were viewed as energy sources, deemed to be sweet and tasty, and considered more or less healthy depending on their sources.

Unawareness of consumption recommendations and the harmful health effects resulted from excessive added sugar intake can be reasons why young adults have a high intake of added sugars. Broad approach for disseminating the risks of excessive sugar consumption and interventions aiming to lower the sugar intake by these populations are strongly recommended.

## Data Availability Statement

The raw data supporting the conclusions of this article will be made available by the authors, without undue reservation.

## Ethics Statement

The studies involving human participants were reviewed and approved by the Human Research Ethics Committee (CEPSH) at the Federal University of Santa Catarina (Under No. 3.063.750). The patients/participants provided their written informed consent to participate in this study.

## Author Contributions

IS and TS was responsible for conceptualization, methodology, formal analysis, investigation, writing—original draft, and writing—review and editing. VR was responsible for conceptualization, methodology, formal analysis discussing, writing—original draft, and writing—review and editing. GB and PU were responsible for conceptualization and writing—review and editing. AF was responsible for conceptualization, methodology, formal analysis discussing, writing—original draft, writing—review and editing, and supervision. RP was responsible for conceptualization, methodology, writing—original draft, writing—review and editing, and supervision. All authors contributed to the article and approved the submitted version.

## Funding

This research was supported by the Brazilian Federal Agency for Support and Evaluation of Graduate Education (CAPES) through a Ph.D. Scholarship (Award No. 41/2018) for TS and by the Brazilian National Council for Scientific and Technological Development (CNPq) through a research productivity scholarship granted to RP (Award No. 305068/2018-0) and Scientific Initiation Scholarship (PIBIC) granted to IS. None of the sponsors influenced the study design, data collection or analysis, manuscript preparation or revision, or publication decisions.

## Conflict of Interest

The authors declare that the research was conducted in the absence of any commercial or financial relationships that could be construed as a potential conflict of interest.

## Publisher's Note

All claims expressed in this article are solely those of the authors and do not necessarily represent those of their affiliated organizations, or those of the publisher, the editors and the reviewers. Any product that may be evaluated in this article, or claim that may be made by its manufacturer, is not guaranteed or endorsed by the publisher.
